# Dissecting structures and functions of SecA-only protein-conducting channels: ATPase, pore structure, ion channel activity, protein translocation, and interaction with SecYEG/SecDF•YajC

**DOI:** 10.1371/journal.pone.0178307

**Published:** 2017-06-02

**Authors:** Ying-hsin Hsieh, Ying-ju Huang, Hao Zhang, Qian Liu, Yang Lu, Hsiuchin Yang, John Houghton, Chun Jiang, Sen-Fang Sui, Phang C. Tai

**Affiliations:** 1 Department of Biology, Center for Biotechnology and Drug Design, Georgia State University, Atlanta, GA, United States of America; 2 State Key Laboratory of Membrane Biology, Center for Structural Biology, School of Life Sciences, Tsinghua University, Beijing China; Centre National de la Recherche Scientifique, Aix-Marseille Université, FRANCE

## Abstract

SecA is an essential protein in the major bacterial Sec-dependent translocation pathways. *E*. *coli* SecA has 901 aminoacyl residues which form multi-functional domains that interact with various ligands to impart function. In this study, we constructed and purified tethered C-terminal deletion fragments of SecA to determine the requirements for N-terminal domains interacting with lipids to provide ATPase activity, pore structure, ion channel activity, protein translocation and interactions with SecYEG-SecDF•YajC. We found that the N-terminal fragment SecAN493 (SecA_1-493_) has low, intrinsic ATPase activity. Larger fragments have greater activity, becoming highest around N619-N632. Lipids greatly stimulated the ATPase activities of the fragments N608-N798, reaching maximal activities around N619. Three helices in amino-acyl residues SecA_619-831_, which includes the “Helical Scaffold” Domain (SecA_619-668_) are critical for pore formation, ion channel activity, and for function with SecYEG-SecDF•YajC. In the presence of liposomes, N-terminal domain fragments of SecA form pore-ring structures at fragment-size N640, ion channel activity around N798, and protein translocation capability around N831. SecA domain fragments ranging in size between N643-N669 are critical for functional interactions with SecYEG-SecDF•YajC. In the presence of liposomes, inactive C-terminal fragments complement smaller non-functional N-terminal fragments to form SecA-only pore structures with ion channel activity and protein translocation ability. Thus, SecA domain fragment interactions with liposomes defined critical structures and functional aspects of SecA-only channels. These data provide the mechanistic basis for SecA to form primitive, low-efficiency, SecA-only protein-conducting channels, as well as the minimal parameters for SecA to interact functionally with SecYEG-SecDF•YajC to form high-efficiency channels.

## Introduction

Most proteins that traverse bacterial cytoplasmic membranes through the Sec-dependent pathway carry a N-terminal hydrophobic signal peptide that is cleaved during, or shortly after, translocation. Biochemical and genetic studies have provided insight into the mechanism of this Sec secretion [1–3]. SecA and its ATPase activity are essential [4–7]. The prevailing model depicts the SecYEG complex as forming the protein-conducting channel with SecA acting as a peripheral ATPase [2, 6], which cycles on and off the cytoplasmic membrane, regulated by SecD-SecF [8, 9]. This model has been reviewed extensively [10–13]. The structure of SecA-SecYEG complex has been reported [14].

There are indications that bacteria can also secrete proteins in the absence of SecYEG both *in vitro* and *in vivo*. Blobel’s group first suggested, and subsequent findings have supported such a SecA-dependent pathway that lacks SecYEG [15–21]. Moreover, it has been shown that SecA integrates into membranes [18, 22–26], that it does not necessarily cycle on and off the membrane during protein translocation [23] and that it is an integral part of the protein-conducting channel [15, 22, 23, 26]. SecA can bind to membranes both at SecYEG high-affinity sites and at phospholipid low-affinity sites [23, 25]. We found that, upon binding to anionic phospholipids, SecA forms ring-like pore structures that may be the core of protein-conducting channels [24, 26, 27]. Indeed, SecA alone can elicit ion-channel activity in liposomes and mediate protein translocation, albeit at low efficiency with no signal peptide specificity [16]. A model for SecA-only protein-conducting channel activity has been proposed [15]. SecYEG adds efficiency and specificity [16, 28]. Purified SecYEG-SecDF•YajC transforms SecA-only liposomes channels into high-affinity channels [29]. These two SecA-dependent channels may serve different functions during the cell growth [17].

*E*. *coli* SecA was reported to be a cytoplasmic protein with 901 amino acid residues [7] (see S1 Table). X-ray structure of soluble SecA has been reported, albeit with a few uncertain regions, especially in the C-terminal region has been determined [30] (see S1 Fig). It is an ATPase with two separate domains, N68 and C34 [31–33] (see S1 Fig) that contain a high-affinity and a low-affinity nucleotide-binding site (NBD) [8, 32, 34] composed of the Walker A and the Walker B motifs that are present in most ATPases. NBD1 contains the Walker A motif at residues #102–109 and the Walker B motif at #198–210 NBDII contains Walker A at #503–511, and Walker B at #631–653 [32, 34]. X-ray analyses of soluble SecA showed that ATP binds to the interface of NBD1 (defined at #1–220), and NBDII (defined at #377–416), and that both domains are required for ATPase activity [35]. The domain containing the intermolecular regulator of ATP hydrolysis (IRA2) is located within a #421–610 region [[36] see S1C Fig] and does not bind to the nucleotide. It regulates ATP hydrolysis, by controlling ADP release [37]. SecA contains other domains including the preprotein-binding domain (PBXD), the helical-scaffold domain (HSD), the helical-wing domain (HWD) and the C-terminal linker domain (CTL) [1, 38]. The C34 fragment has been proposed to be involved in dimerization [39, 40]; it contains IRA1 domain and the regions involved in SecY or SecB interaction as well as a lipid-binding site and a SecB-binding domain [41].

In this study, we characterized the structural and functional roles of the various SecA domains that participate in ATPase activity, interaction with lipids, formation of pore-ring structures, ion-channels, protein translocation activity, and interactions with SecYEG and SecDF•YajC. We used several N-terminal SecA fragments to define which regions are involved in forming functional SecA-dependent channels. Using deletion-truncated fragments of the 901-residue SecA protein and liposomes in the oocytes recordings, we showed that the Helical Scaffold Domains of aminoacyl residues #620–831 are critical for the formation of functional pore channels in phospholipids, and for interacting with SecYEG-SecDF•YajC to gain higher activity.

## Material and methods

### Strains and plasmids

MC4100 is derived from *Escherichia coli* K12 strain (*F- lacU169 araD136 relA- rpsL150 frbB5301 deoC7 ptsF25 thi-)* which was obtained from J. Beckwith [3]; Strain BA13 (MC4100 *supF* ^*ts*^ *trp* ^*am*^ *secA13* ^*am*^ *zch*::*Tn10*) [4] was obtained from D. Oliver. *E*.*coli* strain 773 with *ompA* deletion is a lab stock [42]. Plasmid encoding SecYE_his_G was from F. Duong [43, 44]

### Inner membrane vesicles preparation

Wild-type MC4100 membranes, and OmpA-depleted 773 membranes, were prepared as described [42]. SecA-depleted BA-13 membranes were prepared from *secAts* BA13 mutant. BA13 cells were grown at 30°C to mid-log phase, shifted to 42°C until growth ceased due to SecA depletion. The cells were collected and the BA13 SecA-depleted membranes were prepared by established procedures [42], and subsequently washed with 8 M urea to inactivate residual SecA.

### Liposomes preparation

Liposomes of *E*. *coli* total lipids (Avanti Lipids) were prepared, as described [16]. The lipids were dried by spin vacuum, re-suspended in 150 mM KCl solution/water and sonicated for 3–5 mins until the solution was clear. Preparations were stored at -80°C and thawed for use only once.

### Construction and nomenclatures of N-terminal and C-terminal domain fragments

To construct the C-terminal SecA derivatives, DNA fragments were amplified by PCR (Mastercycler Gradient Pro, Eppendorf) with 5’ primer (TATACATATGCTAATCAAATTGTTAACT) and appropriate 3’ primers using pET5a-SecA. Amplified DNA fragments encoding SecA were cloned into pET5a through *Nde*I and *Bam*HI. Plasmids carrying SecA derivatives were transformed into SecA deficient mutant cells, BL21.19, at 30°C. Some constructs are shown in Supplementary Information S1 Table. In this paper, except for N68 (N-terminal 68 kD protein, referred as N609 in this paper) and C34 (C-terminal 34 kD protein; SecA_610-901_), the domains are defined as N-terminal fragments, thus N350 is SecA_1-350_, N496 is SecA_1-496_, N609 is SecA_1-609_, etc. The C-terminal C30 (SecA_640-901_) and C28 (SecA_654-901_) were similarly constructed.

Some constructs were not stable; to obtain stable clones the C-terminal residues were mutated, thus domains N629*, N639*, N772* and N798* were single mutations at the C-termini (N629D, F639D, D772R, and D798R). The constructs were verified and confirmed by DNA sequencing in the GSU Biology Core Facility.

### Protein purification

*E*.*coli SecA (*EcSecA), its derivatives, and pIMBB28 [36] (for His-N68), were over-expressed from plasmids constructed (see S1 Table) and purified from BL21 (λDE3)/pT7-SecA in *E*. *coli* BL21 (λDE3) as described [4, 16]. The purity of each fragment was >95% as determined by SDS gels. OmpA and pOmpA were prepared, as previously described [16, 22]. SecDF•YajC was expressed from BL21(λDE3) containing plasmid pET543 (obtained from A. Driessen) and purified as described [29]. SecYEG was prepared from pBAD/*secE*_*his*_*YG* (obtained from F. Duong) in C43 strain, as described [16, 45]. Cell-free lysates were passed through a Ni-NTA affinity column (Qiagen), followed by Q-Sepharose cation exchange chromatography (GE Healthcare). SecYEG complex was eluted at 300 to 600 mM NaCl in Tris-HCl, pH 7.9 buffer containing 1% Triton X-100, 10% glycerol and 2 mM DTT, and stored at -80^°^C in the same buffer until use. Protein amounts were estimated from A_280_/A_260_ ratios, and confirmed by Bradford assay as described [16, 29].

### *In vitro* ATPase activity assay

ATPase activity assays were performed with minor modifications, as described previously [46]. For intrinsic ATPase assay, 50 μL reaction mixture contained 1.5 μg of SecA derivatives, 20 μg ovalbumin (optimized for obtaining consistent results), 1.2 mM ATP, 50 mM Tris-HCl (pH 7.6), 20 mM KCl, 20 mM NH_4_Cl, 2 mM Mg(OAc)_2_, 1 mM DTT at 40°C for an appropriate time. For Lipid ATPase assays, 3 μg liposomes was incubated at 30°C. ATPase activity was determined by the release of inorganic phosphate detected by the photometric method [46], absorption was measured at 660 nm (SmartSpec Plus, Bio-Rad Laboratories, Inc.). All assays were performed at least in triplicate.

### *In vitro* protein translocation

Translocation of pOmpA into liposomes or membrane vesicles was conducted as described previously [16]. Unless otherwise indicated, the translocation mixtures in 0.1 mL contained 120 μg of liposomes or 4.5 μg of OmpA-depleted, Urea-washed 773 membranes, 1 μg SecA or 1 μg SecA N831 or 0.7 μg SecA N-terminal variants plus 0.3 μg C34, 0.1 μg SecB and 150 ng substrates pOmpA [16]. The mixtures were incubated at 37°C for 30 min. The translocation mixtures were treated with Proteinase K at 400 μg/ml in ice water for 30 min to remove non-translocated pOmpA, and liposomes or membranes were collected by centrifugation. Translocated proteins were detected by immunoblots, as described previously [16, 29].

### *Xenopus* oocyte injection and whole cell recording

Oocytes were collected and prepared from *Xenopus laevis* (Xenopus Express, Inc) and injected with sample mixtures as described previously [47, 48]; 50 nL sample mixture was injected into oocytes using a Nanoject II injector (Drummond Scientific Co., Broomall, PA). Unless otherwise noted, the amount for each component was 120 ng liposomes, 120 ng SecA, 14 ng pOmpA, 2 mM ATP, and 1 mM Mg^2+^_._ The effective concentration of the reagents in 50 nL injected mixtures was estimated based on an average volume of oocytes of 500 nL. The amount for SecYEG and SecDF•YajC used where indicated was 30 ng for each complex [29]. A two-electrode voltage clamp was used to measure the opening of protein-conducting channels in the oocytes as described previously [16, 47, 49]. Current was recorded for 1 min after 3 hours of incubation at 23°C. The inward and outward currents were recorded to measure the net currents.

### Atomic force microscopy (AFM)

AFM samples were prepared, as previously described [26], with minor modifications: 1 μg of SecA or its derivatives with or without liposomes was mixed by vortex and incubated on ice for 30 min. AFM images were obtained with a MultiMode VIII Scanning probe microscope (Veeco Instrument Inc., Woodbury, NY) using tapping mode at a scan rate of 0.3 Hz. The data were analyzed by image-processing software (Nanoscope v700) according to the manufacturer’s manual.

## Results

### Intrinsic and lipid-stimulated ATPase of SecA domains

**Fig 1:**
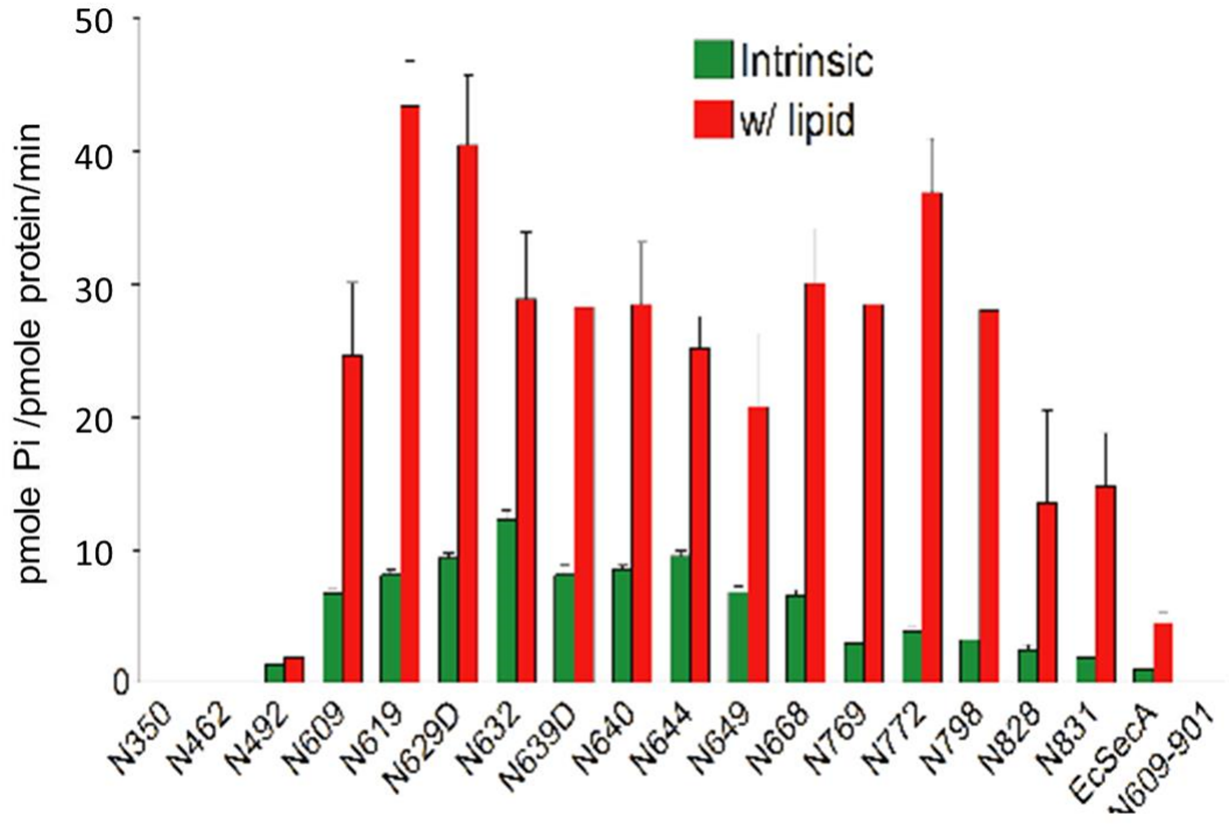
Intrinsic and lipid-stimulated ATPase activities of SecA N-terminal fragments. The assays are as described in Experimental Procedures.

SecA has two separable, soluble fragments: an N-terminal 68 kDa domain (N68; in this study is referred to as N609, which represents N-terminal SecA_1-609_, see Supplement Information S1 Table) which possesses high ATPase activity and the C-terminal 34 kD domain (C34) which has been shown to regulate the ATPase activity [36, 50]. The soluble form of intact SecA possesses low intrinsic ATPase activity that is stimulated by anionic phospholipids, and further by SecYEG with precursors [50]. We constructed a series of N-terminal fragments of SecA to determine the location of ATPase activity, lipid-stimulations and other functions. The intrinsic ATPase has been attributed to the nucleotide binding site I (NBDI), which resides within the first 220 aminoacyl residues of SecA (32, 40, see also Supporting Information S1C Fig). SecA N350 which forms partial, pore ring-structures [15] has little ATPase activity that could be stimulated by phospholipids (Fig 1).

We previously found that the first 25-aa N-terminual residues and an EM domain of SecA are necessary for membrane insertion [51, 52], and lipid-ATPase activity [15, 52]. We found that two forms of SecA exist in the membrane [15, 29]. One form (SecA_M_) that occurs in the presence of phospholipids contains the central M48 tryptic fragment at Glu_361._; the other (SecA_S_) contains a 68-kDa (N609) fragment, which is part of a domain in the soluble SecA [33]. There are two tryptic lipid-specific domains: an N-terminal 36-kDa (N36) and a middle 48-kDa (M48) domain present within the SecA_M_ form [15, 23]. We examined whether various domains of SecA (for domain/fragment names, see nomenclature used in Procedures and S1 Table) have ATPase activity that can be stimulated with phospholipids. Of the SecA_S_ domains (N609 and C34) both of which incorporate into membranes [31], N609 has the higher ATPase activity. Of the SecA_M_ domains, neither N350 nor M48 -starting at Glu_361_ [15]- has detectable intrinsic ATPase activity, despite each’s having an ATP-binding domain (S1 Fig). Thus the ATPase domain, in addition to NBDI, lies between N350 and N609 (Fig 1). It has been reported that two ATP-binding domains are required to promote translocation [32, 33], and the X-ray structure of SecA indicated that ATP binds to the interface of two NBD sites [35]. We constructed a series of N-terminal SecA domains/fragments associated with C-terminal deletion to determine the potential location of intrinsic ATPase and lipid-stimulated ATPase activities. While most purified fragments interacted with lipids, the critical N-terminal domain for ATPase was around N492, which has 30% higher intrinsic activity than that of the intact SecA. Even so, the lipid-stimulated ATPase activity was significantly less, when compared to intact SecA (1.4-fold compared to 4-fold with intact SecA under the conditions tested). The intrinsic ATPase activity of the N-terminal fragments increased up to N632 (Fig 1). The soluble N609 domain had a high intrinsic ATPase activity that increased when interacting with lipids (the reported lack of stimulation by lipids is due to the presence of His-N609, data not shown). Maximal lipid-stimulated ATPase activity was found to be around domain-fragment N619; successive fragments remained high up to N798 (Fig 1). ATPase activities decreased from N798—N831, with the intact SecA having the lowest activity of all (Fig 1), presumably due to regulatory inhibition by interactions with the C-terminal domain [36, 53].

### Formation of ring-pore structure with liposomes observed in AFM

**Fig 2:**
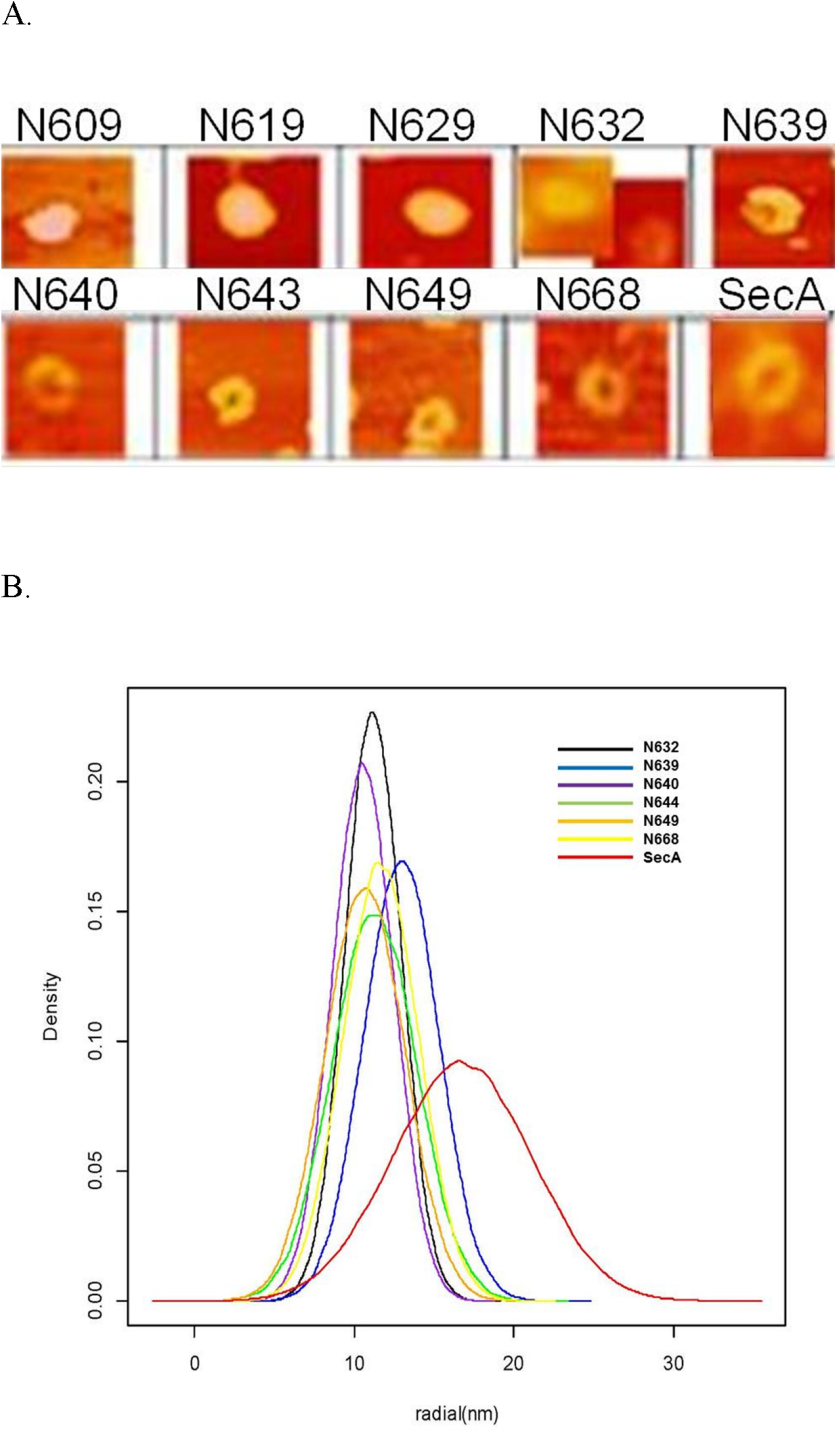
AFM structure image of SecAs. (A) AFM images formed by SecA/SecA fragments. (B) Normal distribution of radius of ring-like structures formed by SecA/SecA N-fragments. Plots were generated by R program, adopted from http://www.r-projet.org (a free software for statistical computing and graphics), based on 100 random samples with similar means and variances of each measured datum. The efficiency of forming pore-ring structure for N640 was about 40%, for N643 or larger >70%, and for N668 >90%.

SecA forms pore-ring structures [26] when interacting with anionic phospholipids. The two lipid-specific domains N350 and M48 form partial ring structures in the presence of phospholipids that are smaller than the complete SecA ring-structure [15]. As the length of SecA fragments was extended beyond N350, the partial pore-structure was lost at around N462; soluble fragments N609—N628 individually interact with lipids and have high ATPase activity, but form no pore structures (Fig 2A). Pore-ring structures could be observed with fragment N639 and larger (Fig 2A), but higher efficiency with N640-N668 with liposomes. These pore structures were different from the partial pore structures observed with N350 or M48 [15]. However, such structures were smaller than those with the full SecA (Fig 2B).

### SecA-only ion-channel and protein translocation activity

**Fig 3:**
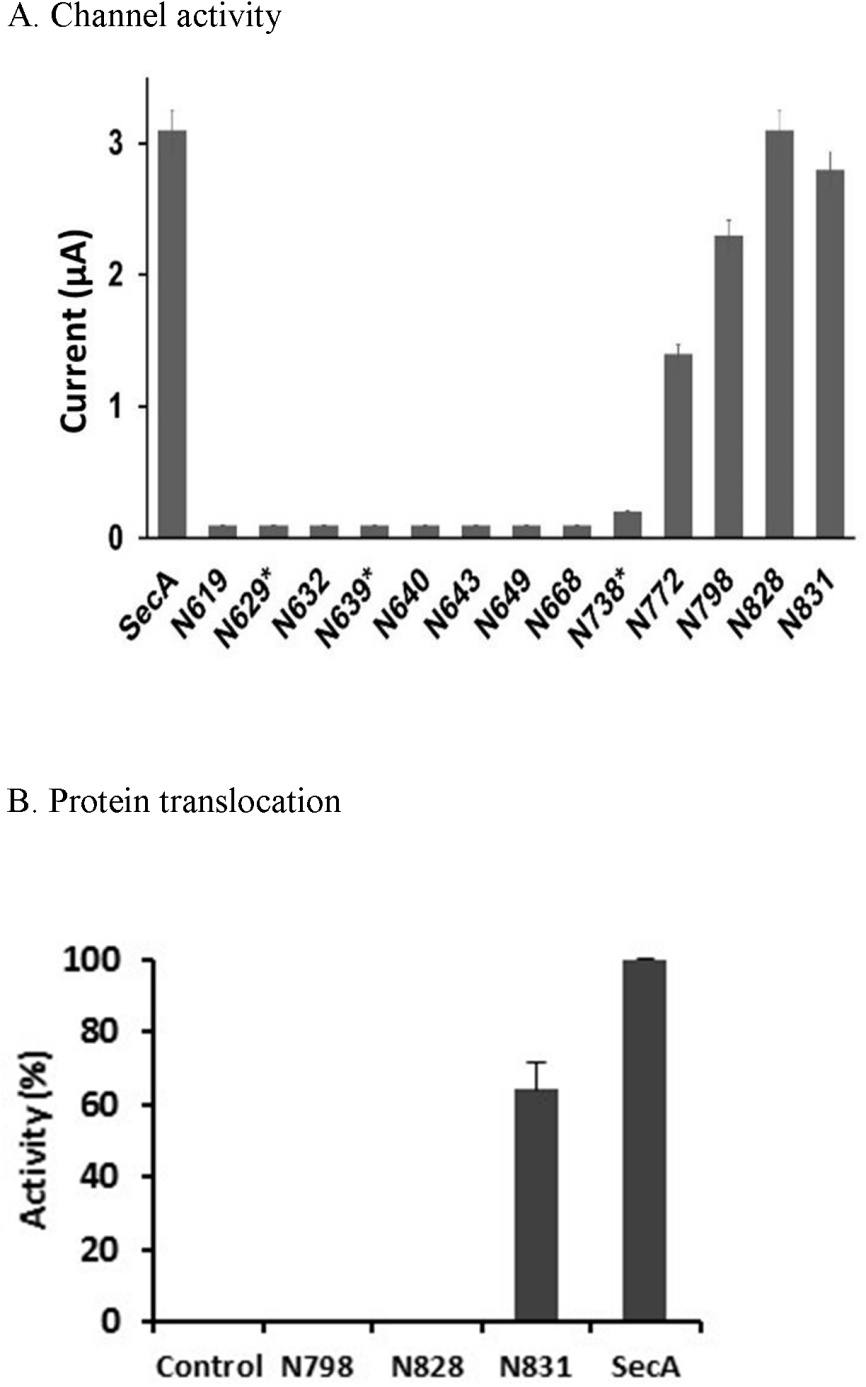
Function of SecA-only liposomes. (A) Channel activity. (B) Protein translocation. Assays as described in Experimental procedures.

We previously identified a SecA-only protein-conducting channel activity in oocytes *and in vitro* protein translocation, and determined its characteristics and the differences from SecA-dependent channels containing SecYEG [16]. We proposed that SecA contains two binding sites for membranes and protein translocation: one with low-affinity phospholipids SecA-only and the other with high-affinity SecYEG [15, 16, 28]. SecA-only channels are less efficient and require additional ATP to elicit even low levels of channel activity [16]. In this study, we determined the SecA-only channel activity of various N-terminal fragments of SecA when associated with liposomes in the oocytes for ion channel activity. The the N350- or M48-liposomes, which form only partial ring-structures [15], had no channel activity (data not shown). Even though fragments N639-N668 formed relatively small ring-pore structures with liposomes, they had no ion channel activity. N738-liposomes, however, began to show a little channel ion current activity with N772-liposomes demostrating about 50% channel activity (with a lower expression rate), while N798-liposomes possessed substantial channel activity (Fig 3A). N828 and larger fragments showed almost the full channel activity of intact SecA-liposome channels.

In addition to eliciting ion channel activity, SecA-liposomes promote protein translocation [16]. We therefore determined whether there is any correlation between SecA-only ion-channel activity and protein translocation activity. In contrast to the channel activities elicited by fragments larger than N772, there was little to no translocation of pOmpA precursors into liposomes up to domain N828; with N831 being the lowest SecA fragment tested that promoted detectable translocation in liposomes (Fig 3B). The 3 residues SecA_828-831_ form part of a hydrophobic helix (S1D Fig), and appeared to be critical for SecA-only channels to translocate proteins.

### SecA domains interacting with SecYEG-SecDF•YajC for channel activity and protein translocation

**Fig 4:**
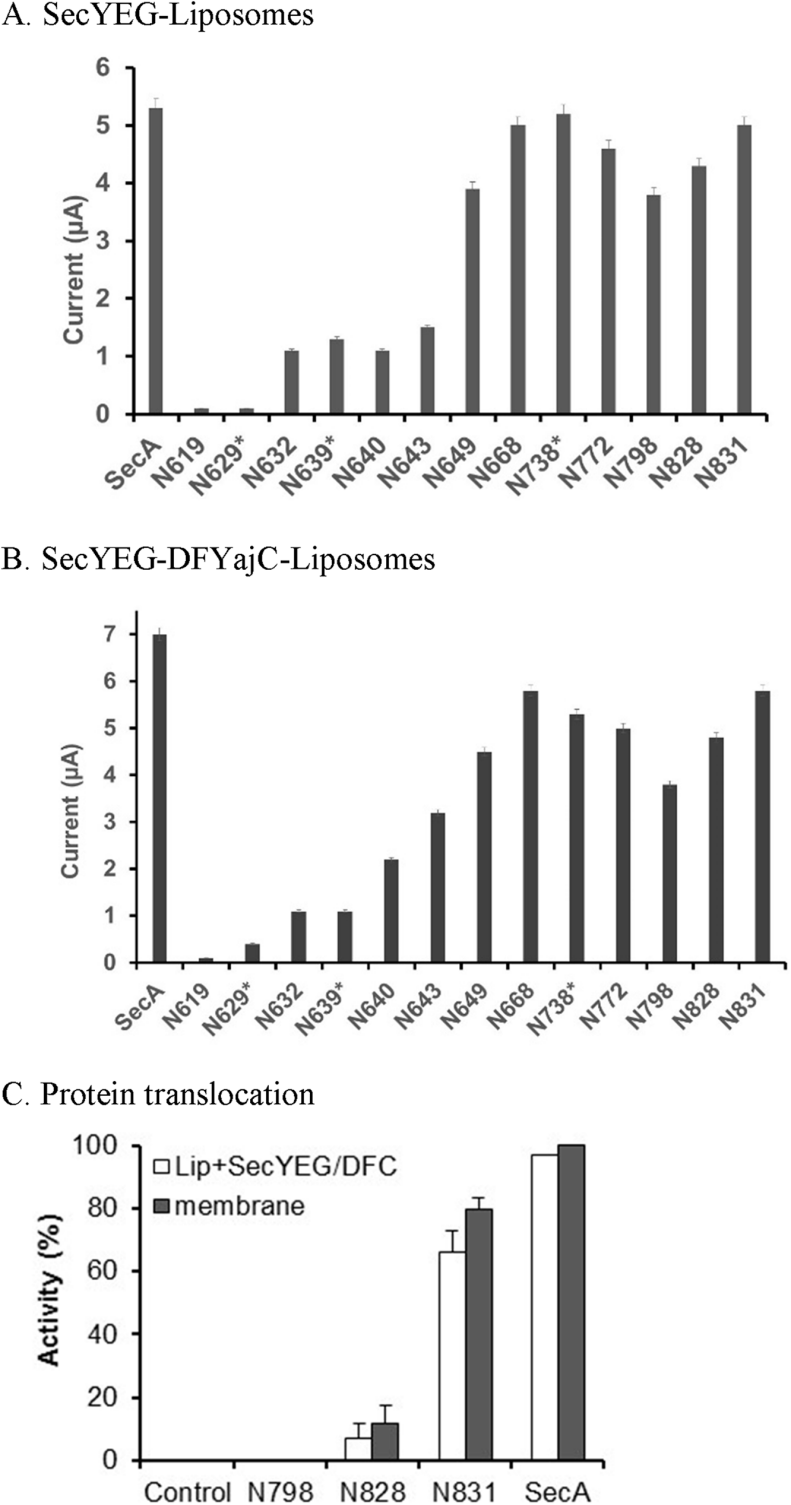
Function of SecA-liposomes with SecYEG/SecDF-YajC. (A) SecA-domain-SecYEG channel activity. (B) SecA-domain with SecYEG-SecDF-YajC Channel activity. (C) Translocation activity in membranes: 1 μg SecA/fragment and 4.5 μg urea treated OmpA-depleted 773 membranes or 120 μg liposomes were used. The translocation in 100 μL were conducted under 37C for 30 min and analyzed as in Experimental procedures.

SecA-SecYEG-SecDF•YajC channels are specific and efficient [16]. We further defined the critical SecA domains’ interactions with SecYEG-SecDF•YajC for ion channel and translocation activity (Fig 4). There was no channel activity in N350, N609, and N619 in the presence of liposomes containing SecYEG or SecYEG-SecDF•YajC, the latter two having been previously shown to possess no channel activity by themselves [16]. The liposomes of N629*, N639* and N640 fragments when complexed with SecYEG had some channel activity, though with less expression efficiency (Fig 4A). There were substantial channel activities for N643-N649 fragments with SecYEG and SecDF•YajC, indicating that the critical SecA residues that interact with SecYEG and SecDF•YajC for channel activity may be located in the SecA_643-649_ aminoacyl residues. The N668 domain, which by itself has no channel activity with liposomes, has full channel activity with SecYEG and SecYEG-SecDF•YajC (Fig 4A and 4B). Channel activity was similarly observed with domains N643, N649, or N668 together with SecA-depleted membranes (Table 1, and data not shown). Although ion channel activity was restored by the reconstitution of the SecA domain fragments with SecYEG-SecDF•YajC, ionic current and expression efficiency in the oocytes were slightly less effective when compared to the SecA-depleted membranes containing SecYEG-SecDF•YajC.

**Table 1:** Summary of critical SecA domains for structure and function with lipids.

SecA Domains	ATPase	AFM Pore	Channel Activity, μA			% pOmpA Translocation		
			Lipo	+YEG	Memb.	Lipo	+YEG	Memb
N609-N619	+++	-	0	0	0	0	0	0
N629-N632	+++	+/-	0	0.4	0.6	ND	ND	ND
N639*	+++	**++**	0	1.1	1.8	ND	ND	ND
N640	+++	+++	0	2.2	**5.0**	ND	ND	ND
N643	+++	+++	0	3.2	**5.7**	ND	ND	ND
N649-N668	+++	+++	0	**5.8**	6.9	0	0	0
N772-N798	+++	ND	**2.3**	ND	ND	0	0	0
N828	++	ND	**3.1**	ND	ND	0	**7.6**	**11.8**
N831	++	+++	2.8	5.8	6.0	**18.8**	71.2	79.5
SecA	+	+++	3.1	7.0	6.8	30.0	97.0	100

Purified truncated SecA domain fragments were tested for lipid-stimulated ATPase, pores structure formation by AFM, and for channel activity in oocytes (n = 30) and protein translocation (n = 6) with pOmpA in liposomes (Lipo), with SecYEG-SecDF•YajC (+YEG) and SecA-depleted membranes (Memb) which contain all native membrane proteins. For clarity, only the relevant data are shown. ND: Not determined.

In order to determine the ion current activity of various domain-fragments with SecYEG and SecYEG-SecDF•YajC we extended the C-terminual amino acid residues to fragments N738*, 772, 798*, 828, and 831. Having shown that the fragment N668 had full ion channel activity with SecYEG-liposomes, increasing the length of SecA fragments to N738*, N772 and then N798* resulted in a significant decrease in channel activity before it was regained in fragments N828 and N831 (Fig 4A and 4B), N831 was able to fully express channel activity, indicating the special function of residues 828–831 which form part of the third helix (S1D Fig). Thus, while amino acids #668–798 appeared to be important for liposomes interacting with SecYEG; their activity is enhanced by residues 828–831.

In view of the conformational changes of SecA that are induced by phospholipids, we tested and correlated various SecA domains interacting with liposomes for their pOmpA translocation activity with SecYEG and SecDF-YajC. As noted earlier, there is no translocation activity with liposomes alone with SecA domains smaller than N828 (Fig 3B). Addition of SecYEG-SecDF•YajC did allow some translocation activity for SecAN828 (~10%), but addition of SecYEG-SecDF•YajC with SecAN831 increased translocation greatly, almost to the same extents as with the full length SecA and intact membranes (Fig 4C). These data again indicate the importance of the SecA_828-831_ region for interacting with SecYEG-SecDF•YajC and promoting protein translocation.

### Complementation of inactive N- and C-SecA framents for structure and functions

Our data indicate that the SecA aminoacyl residues #609–831 are critical for pore-formation, channel activity, protein translocation and interaction with SecYEG. The X-ray structures [40] show that this domain contains 3 helices (S1D Fig): the long Helical Scaffold Domain (#619–668), which consists of two subdomains, HSDI (#619–639) and HSDII (#640–668), the latter of which forms the pore-structure, and possesses channel activity. HSDI has no such capability, but possibly provides the ability to reconstitute active channels through interactions with otherwise inactive C-terminal domains. The C34 domain (SecA_610-901_), which contains the 3 helices and the extreme C-terminal 70 aminoacyl residues, has been characterized for its ability to down-regulate ATPase activity [IRAI; [8, 36]]. We examined the ability of this domain to complement the various non-functional N-terminal domains for pore formation, channel activity and protein translocation.

#### SecA-liposome domain reconstitution

**Fig 5:**
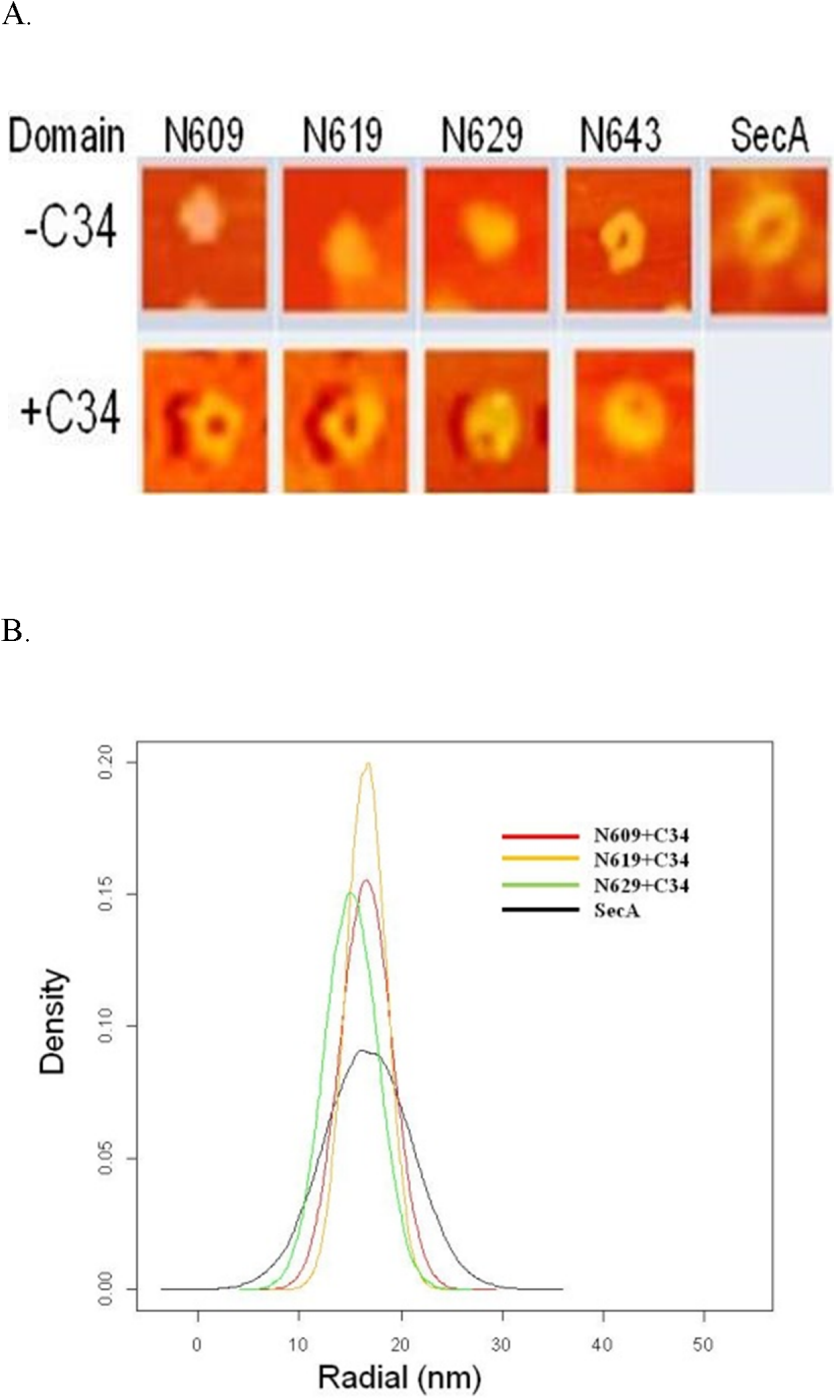
Complementation of SecA N-domains for AFM ring-structures. Same amounts of proteins were used: molar ratios of C34: N-fragments were about 2:1. Full length SecA was used for comparison. (A) AFM images with mean sizes and SD. (B) Size distribution as analyzed in Fig 2B. No complementation of pore structures with ovalbumin instead of C34 (data not shown).

**Fig 6:**
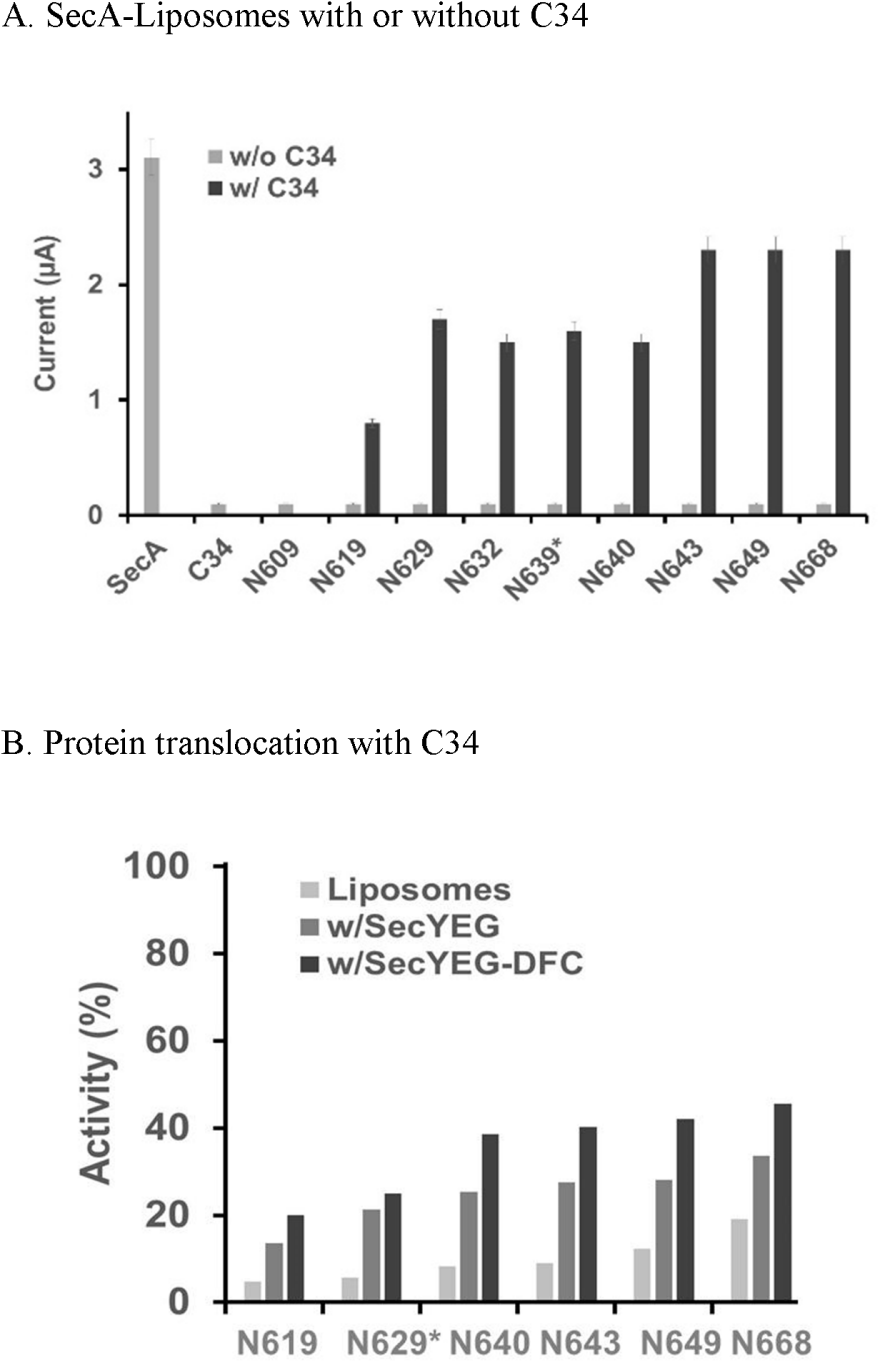
Functional complementation of SecA fragments with C-terminal fragment C34. (A) Channel activity in SecA domains reconstituted with C34, SecA_610-901_. (B) Translocation activity in SecA domains reconstituted with C34. N609 together with C34 have no activity, and could serve as internal negative controls. The reactions were conducted with liposomes with SecYEG or SecYEG-SecDFC as indicated. 100% translocation activity is SecA with SecYEG-DFC.

Fragments N609 and N619, which by themselves do not, form pore structures in liposomes (Fig 2), do so in combination with C34 (SecA_610-668_), though with low efficiency; they are similar in size to those formed by intact SecA (Fig 5A and 5B), N609 with C34 has no ion channel activity, which serves as internal negative control for complementation with C34 (Fig 6A). N619 and C34 overlaps residues #610–619 and together have only a little ion-channel activity; reconstitution of C34 with fragments N629-640 yields more. Reconstitution with N643-N668 yields as much channel activity as those with SecA-only-liposomes (Fig 6A). Protein translocation activities of C34 also increased with longer N-terminal regions in SecA-only reconstituted liposomes, reaching more than 60% of the activity of intact SecA (Fig 6B). (Such translocation activity is comparable to that with SecYEG-SecDFC which is about 50% of intact SecA activity, Fig 6B).

**Fig 7:**
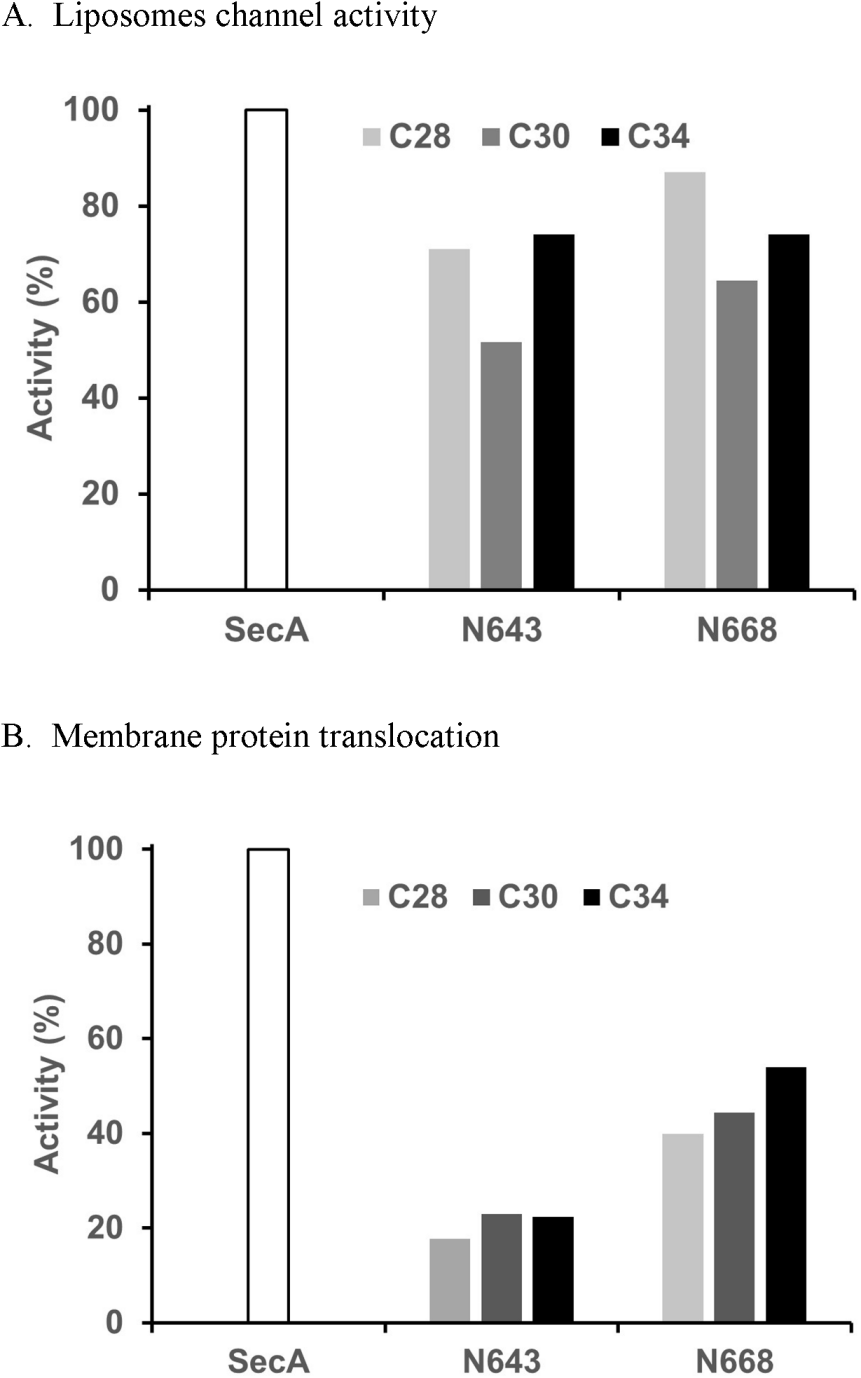
Complementation with C-terminal domains for activities. (A) Liposomes channel activity. N643 or N668 alone has no channel activity (B) Membrane protein translocation. C34 fragment: SecA_610-901_. C28 fragment: SecA_662-901_. C30 fragment: SecA _640–901_. None of SecA fragments alone has any translocation activity.

The C34 domain overlaps various N619-N668 fragments, e.g. it overlaps almost 60 aminoacyl residues of N668. We examined the interaction of N668 with smaller C-terminal constructs, including C30 (SecA_640-901_) and C28 (SecA_654-901_). Interactions with both C-terminal constructs yielded active SecA-only channel activity; the interaction with C28 (with only a 13 aminoacyl residue overlap) yielded almost complete SecA-alone channel activity (Fig 7A). These findings show that the region SecA_610-653_ of C34 is not necessary for complementation, and that small overlapping regions yield channels activities equivalent to that of SecA-alone.

#### SecA fragments reconstitution with SecYEG-SecDF•YajC

We examined interactions of SecA fragments and liposomes with SecYEG-SecDF•YajC, and found some synergistic increases in channel activities with fragments up to N640. Domains in HSDII #643–668, which had high channel activity with C34, did not increase channel activity (data not shown), indicating that large overlaps in the HSD helix do not enhance interactions with SecYEG-SecDF•YajC or with intact membranes. In contrast, translocation activities of reconstituted C34 and N-terminal SecA domains were much more pronounced (Fig 6B). There were substantial translocation activities for fragments N619- and N629- liposomes (but not N609) with SecYEG-SecDF•YajC and C34, while N640-N668 reached maximal levels when reconstituted with SecYEG-SecDF•YajC and C34, attaining translocation activity of about 50% activity found for intact SecA (Fig 6B).

We also examined the translocation effects of C-terminal overlapping residues in interaction with native SecA-depleted membranes containing SecYEG-SecDF•YajC. There were translocation activities observed in all C34, C30 and C28 fragments in combination with N643 and N668, with the longer C34 having a slightly higher translocation activity (Fig 7B). There was no translocation activity when combined with no overlapping region, i.e. N609. The overlapping regions, therefore, did appear to have some effects on translocation.

## Discussion

*E*.*coli* SecA, which contains 901 amino acids, interacts with multiple components of the Sec protein translocation system. In this study, we defined which of the SecA domains are important for interaction with lipids, for ATPase activity, for ring-pore formation, for ion channel activity, and for protein translocation. We also defined which regions interact with SecYEG-SecDF-YajC (Table 1). In liposomes alone, SecA_1-831_ is minimally required for protein translocation;, SecA_1-772_ for ion channel activity, SecA_1-639_ for pore formation, and SecA _1–609_ for ATPase activity. SecA_1-649_ and SecA_1-828_ interact with SecYEG-SecDF•YajC yielding higher efficient channel and translocation activities, respectively (Table 1). Thus, we have established which SecA domains are required for each of the two SecA-dependent protein translocation pathways: the low-affinity, low-efficiency, SecA-only protein-conducting channels, and the high-affinity, high-efficiency SecA-SecYEG-SecDF•YajC protein-conducting channels [15, 16, 29]. This work provides evidence for which domains are critical for structures and function, particularly the three helical domain region of SecA (SecA_619-831_), which appears to coordinate key features of these processes (S1 Fig and Table 1).

The soluble N609 domain has high intrinsic ATPase activity and can interact with lipids to yield higher activity. The soluble N609 domain containing NBDI and probably a partial NBDII [32, 34] also has high intrinsic ATPase activity which increases upon interaction with lipids. N-terminal deletions dramatically affect this activity [39, 52]; and a point mutation, F586L, lost the lipid-stimulated ATPase activity of N619 (Data not shown). The long helix (S1C and S1D Fig), which contains a putative NBD II Walker B site (#631–653, Ref 32; S1C Fig), drastically changes the interaction with lipids. The region of SecA from residues #621–831 is particularly important for forming structures and functions with lipids. It consist of three helices (S1C and S1D Fig): 1) the helical Scaffold Domain HSD (N619-668), 2) HSDI, N621-640 which forms fully functional channel activity with membranes, HSDII, N641-668 with SecYEG-SecDF•YajC, HWD (N762-782) and RAI (N810-831). Table 1 highlights which domains that are critical for forming pore structures with liposomes (N629), for channel activity with membranes (N640), and for interactions with SecYEG (N649), SecDF•YajC (N643), liposome-only channels (N798) and translocation (N829). These regions appear to be critical for forming pore structures in the liposomes. N640 and larger domains up to N668 form small ring-pore structures (Fig 2), which by themselves have no ion channel activity in the liposomes (Table 1). Domains between N649-N668 yield higher ion channel activity when reconstituted with SecYEG-SecDF•YajC (Table 1). In contrast, differences in protein translocation activities are relatively small (#828–831) for SecA-only and SecA-SecYEG-SecDF•YajC channels.

SecA can exist as a soluble hydrophilic protein, yet it has many regions that interact with lipids, allowing it to integrate into liposomes and membranes [15, 22, 23, 26, 31, 54, 55]. Previous studies revealed several sites for liposomal binding: one is located within the region encompassed by the amino acids #1–22 [52], #101–118 [51] and two more [25] that form partial pore structures [15, 22]. It has been proposed that SecA contains two lipids binding-sites located at both the N-terminal (1–100) and C-terminal (600–900) ends of the protein [31, 56, 57]. The C-terminal has been reported to contain lipid binding sites [41, 55, 58, 59]. Keller [60], predicted several SecA-lipid binding sites (SecA_1-21,_ SecA_14-33,_ SecA_43-60,_ SecA_66-90,_ SecA_108-125,_ SecA_370-395_, SecA_400-488_ SecA_593-614_ SecA_635-660_ SecA_804-822_ SecA_865-882,_ SecA_877-895_). Thus, many SecA domains interact with lipids and, in so doing, affect ATPase activity, structure and function. These interactions are dynamic. Our results are compatible with these previous findings, and define more precisely which regions determine ring-pore-structure formation, that form ion channels, as well as which regions interact with SecYEG-SecDF•YajC mediating protein translocation.

There are two membrane-binding sites in SecA in the membranes: one high-affinity, with SecYEG, and the other low-affinity with liposomes alone [2, 16, 32, 33, 61, 62]. Translocation at each site is SecA-dependent and their efficiency and specificities vary with SecA’s interaction with SecYEG-SecDF•YajC, which SecA dependent [16, 28, 29]. Using SecA-deletion fragments, we are able to map the critical domains responsible for the channel activity of SecA-only and/or SecA-SecYEG-SecDF•YajC liposomes. SecA amino acid residues #640–649 are critical for SecYEG interactions (Table 1; S1D Fig). These observations are consistent with earlier site-directed, *in vivo* photo-crosslinking studies [63–65] that identified several amino acids including #640 and #661 of SecA as being essential for SecYEG interactions. The crystal structures of SecA-SecYEG complex also indicates the importance of the SecA long HSD interacting with SecY [14], and #640–661 are structurally critical for SecY interactions. Our data further support the importance of this region for the SecA-SecYEG functional interactions. However, the SecA-SecYEG-SecDF•YajC liposomes failed to generate ionic currents as high as those observed with *E*. *coli* membranes containing SecYEG-SecDF•YajC, an inability that was also observed earlier with pPhoA [28]. This may suggest that the translocation machinery components in the membrane have more than just SecA and SecYEG-SecDF•YajC. The crystal structure of SecDF has been reported [66]. SecDF is involved in the stabilization of SecYEG, and maintenance of a proton motive force to enhance protein translocation [44, 66–69]. Other accessory proteins also play important roles in translocation channel activity. Randall’s group has identified several SecA domains that interact with the molecular chaperone SecB [62, 63].

We previously proposed that there are two SecA-dependent protein-conducting channels: the SecA-SecYEG-SecDF•YajC high-affinity protein conducting channels, and low-affinity, less specific SecA-only channels [15, 16, 28, 29]. This work supports that proposal and defines which SecA domains interact with lipids to form SecA-only channels. These interactions provide a basis for there being different structures and functions for SecA-only channels. The domains required for pore formation are different from those required for the ion-channel and protein-translocation activities. Identification of different domains for ATPase, pore structure, channel activity and protein translocation support the idea that SecA functions at least as a dimer [15, 27, 39, 56, 70, 71] and refs. therein) in the SecA-only channels. Interactions with SecYEG-SecDF•YajC require fewer SecA residues. This work further refines the SecA domains that interact with SecYEG-SecDF•YajC to form high-efficient and signal-specific channels. Such (SecA)-SecYEG channels are probably more important during the exponential phase of growth [17, 28]. There is a notable surfeit of SecA in stationary phase [17]. Although it is not clear how our findings relate to in vivo, we speculate that SecA-only channels play a physiological role in stationary phase [16, 17, 29], as well as secretion of cytoplasmic proteins that lack a signal peptide [72]. SecA is required for biogenesis and co-translational insertion of certain membrane proteins [73–76]. SecA binds to ribosomes [77, 78] and competes (in a mutually exclusive manner) with SecYEG for such attachment [79]. Ribosomes might associate with more with SecA-only channels than with SecYEG channels for co-translational secretion, especially for proteins that lack signal peptides. Many cytoplasmic membrane proteins lack cleavable signal sequences, and so may not be recognized by SecYEG channels, the components of which also lack a signal peptide, and presumably be placed in the membrane through SecA-only channels. Thus, SecA-only channels may involve co- and post-translational translocation, Their competitive binding to ribosomes may explain why there is more SecA than SecYEG in bacterial cells.

## Supporting information

S1 Table E. coli SecA protein sequence and the construct domain sequences. Protein sequence of EcSecA derived from nucleotide sequence [33]. The residues for three critical helices (see S1C and S1D Fig) are color high-lighted. The various domains were constructed, over-expressed and purified. The extreme C-termini of N629 and N639 were mutated with D for stable expression of the constructed domains. The domains are presented as residues from SecA, thus N609 refers to SecA1-609, or C-terminal domains, C34 refers to SecA610-901.
(DOCX)

S1 Fig Structure and hydrophobicity of E.coli SecA. **A**. Hydrophilicity plot of SecA sequence [34] (from proScale, ExPASy Bioinformatics Resource Portal).
**B**. Multiple lipid-binding domains [15, 22, 25, 31, 33, 51, 52]. N68 (N609), C34 (# 610–901), N350 and M48 (SecA361-798) are trypsin fragments; the latter 2 are lipid-specific [15, 22].The lower bars: known and predicted SecA lipid-binding domains [60]: (SecA1-21, SecA14-33, SecA43-60, SecA66-90, SecA108-125, SecA370-395, SecA400-488 SecA593-614 SecA635-660 SecA804-822 SecA865-882, SecA877-895).
**C**. Linear presentations of critical SecA domains and Subdomains: Nucleotide binding domain (NBDI and II NBDI: A1:102–109; B1:198–210; NBDII: A2:503-511B2: 631–653 [34]; Preprotein binding domain); α-helical wing domain (HWD; a long helical scaffold domain (HSD) #621–668 containing two subdomains:# 621–640 (Orange) important for forming pores and 641–668 (Green) critical for SecYEG interaction [14, 32, 64].1 deep-blue helix RA1 (# 756–788) for channel activity; 1 red-helix (#802–829) for protein translocation.
**D**. Simulated EcSecA X-ray ribbon structure [30]. (Protein Data Bank #2FSF; only #12–831 available; Pink, N-terminal helix #1–11; precursor binding domain, (PPXD) and C-terminal domains CTD are not available). E. coli SecA monomer 3D ribbon structure predicted by Pymol software (The PyMOL Molecular Graphics System, Version 1.7.4 Schrödinger, LLC.).
Left panel: SecA ribbon structure of 3 highlighted critical helix domains. The 3 helical domains #609–831 form hydrophobic interactions and are probably critical for SecA functions. Right panel: Zoom in simulated 3D structure of three critical helices with interacting hydrophobic residues. Green: 651-667aa, Blue: 762-782aa, and Red: 810-829aa. The hydrophobic amino acids are marked on three helices. The I695 is close to F811, M814, L815 and L818 after 180 degree turn on 3D structure.
(DOCX)
