# Supplementary material for: Dissecting structures and functions of SecA-only protein-conducting channels: ATPase, pore structure, ion channel activity, protein translocation, and interaction with SecYEG/SecDF•YajC
Source: PLoS One. 2017 Jun 2;12(6):e0178307. doi: 10.1371/journal.pone.0178307 (PMC5456053; doi:10.1371/journal.pone.0178307)
Supplement: S1 Table — Protein sequence of EcSecA derived from nucleotide sequence [33]. The residues for three critical helices (see S1C and S1D Fig) are color high-lighted. The various domains were constructed, over-expressed and purified. The extreme C-termini of N629 and N639 were mutated with D for stable expression of the constructed domains. The domains are presented as residues from SecA, thus N609 refers to SecA1-609, or C-terminal domains, C34 refers to SecA610-901. (DOCX) [file pone.0178307.s001.docx]

**S1 Table. E. coli SecA protein sequence and the construct domain sequences.** Protein sequence of EcSecA derived from nucleotide sequence [33]. The residues for three critical helices (see S1C and S1D Figs.) are color high-lighted. The various domains were constructed, over-expressed and purified. The extreme C-termini of N629 and N639 were mutated with D for stable expression of the constructed domains. The domains are presented as residues from SecA, thus N609 refers to SecA1-609, or C-terminal domains, C34 refers to SecA610-901.


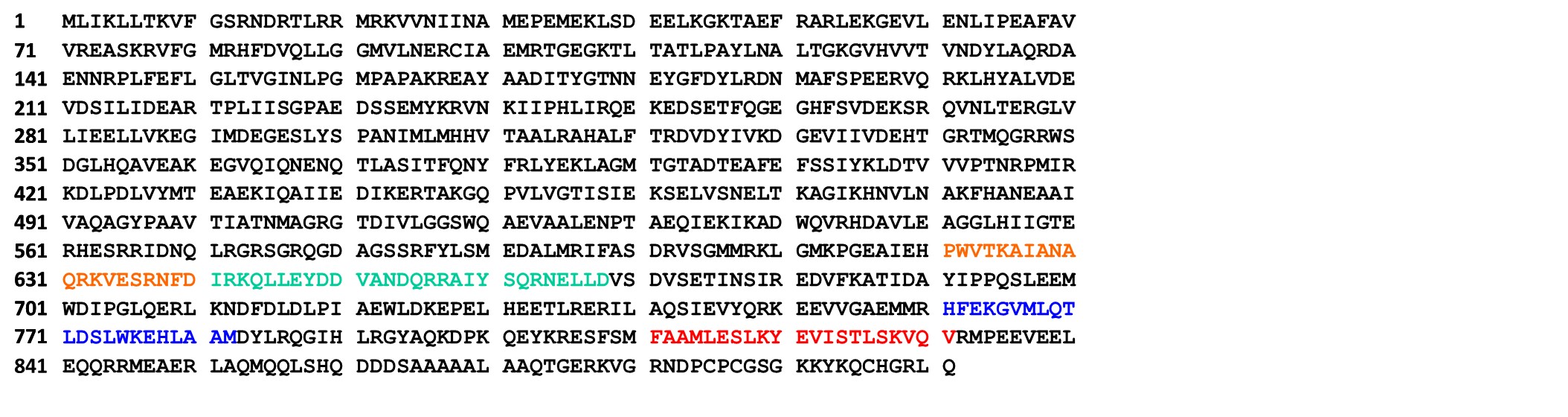


**EcSecA**  ^1^M----**^600^**drvsgmmrkl gmkpgeaieh pwvtkaiana qrkvesrnfd irkqlleydd vandqrraiy sqrnelldvs**^670^**--------Q**^901^**

**Domain Constructs**

**N609** ^1^M------- drvsgmmrk^609^

**N619**  ^1^M------- drvsgmmrkl gmkpgeaie

**N629D**  ^1^M------- drvsgmmrkl gmkpgeaieh pwvtkaia**D**

**N632**  ^1^M------- drvsgmmrkl gmkpgeaieh pwvtkaiana qr

**N639D** ^1^M------- drvsgmmrkl gmkpgeaieh pwvtkaiana qrkvesrn**d**

**N640**  ^1^M------- drvsgmmrkl gmkpgeaieh pwvtkaiana qrkvesrnfd

**N643** ^1^M------- drvsgmmrkl gmkpgeaieh pwvtkaiana qrkvesrnfd irk

**N649**  ^1^M------- drvsgmmrkl gmkpgeaieh pwvtkaiana qrkvesrnfd irkqlleydd

**N657** ^1^M------- drvsgmmrkl gmkpgeaieh pwvtkaiana qrkvesrnfd irkqlleydd vandqrr

**N668** ^1^M------- drvsgmmrkl gmkpgeaieh pwvtkaiana qrkvesrnfd irkqlleydd vandqrraiy sqrnelld

**C34 (_610-901)_** L gmkpgeaieh pwvtkaiana qrkvesrnfd irkqlleydd vandqrraiy sqrnelldvs**^670^** –------Q**^901^**

**C30** (**_640-901)_**  d irkqlleydd vandqrraiy sqrnelldvs**^670^** -------Q**^901^**

**C28 (_654-901)_**  dqrraiy sqrnelldvs**^670^** ------- Q**^901^**
