# Supplementary material for: Dissecting structures and functions of SecA-only protein-conducting channels: ATPase, pore structure, ion channel activity, protein translocation, and interaction with SecYEG/SecDF•YajC
Source: PLoS One. 2017 Jun 2;12(6):e0178307. doi: 10.1371/journal.pone.0178307 (PMC5456053; doi:10.1371/journal.pone.0178307)
Supplement: S1 Fig — A. Hydrophilicity plot of SecA sequence [34] (from proScale, ExPASy Bioinformatics Resource Portal). B. Multiple lipid-binding domains [15, 22, 25, 31, 33, 51, 52]. N68 (N609), C34 (# 610–901), N350 and M48 (SecA361-798) are trypsin fragments; the latter 2 are lipid-specific [15, 22].The lower bars: known and predicted SecA lipid-binding domains [60]: (SecA1-21, SecA14-33, SecA43-60, SecA66-90, SecA108-125, SecA370-395, SecA400-488 SecA593-614 SecA635-660 SecA804-822 SecA865-882, SecA877-895). C. Linear presentations of critical SecA domains and Subdomains: Nucleotide binding domain (NBDI and II NBDI: A1:102–109; B1:198–210; NBDII: A2:503-511B2: 631–653 [34]; Preprotein binding domain); α-helical wing domain (HWD; a long helical scaffold domain (HSD) #621–668 containing two subdomains:# 621–640 (Orange) important for forming pores and 641–668 (Green) critical for SecYEG interaction [14, 32, 64].1 deep-blue helix RA1 (# 756–788) for channel activity; 1 red-helix (#802–829) for protein translocation. D. Simulated EcSecA X-ray ribbon structure [30]. (Protein Data Bank #2FSF; only #12–831 available; Pink, N-terminal helix #1–11; precursor binding domain, (PPXD) and C-terminal domains CTD are not available). E. coli SecA monomer 3D ribbon structure predicted by Pymol software (The PyMOL Molecular Graphics System, Version 1.7.4 Schrödinger, LLC.). Left panel: SecA ribbon structure of 3 highlighted critical helix domains. The 3 helical domains #609–831 form hydrophobic interactions and are probably critical for SecA functions. Right panel: Zoom in simulated 3D structure of three critical helices with interacting hydrophobic residues. Green: 651-667aa, Blue: 762-782aa, and Red: 810-829aa. The hydrophobic amino acids are marked on three helices. The I695 is close to F811, M814, L815 and L818 after 180 degree turn on 3D structure. (DOCX) [file pone.0178307.s002.docx]

**S1 Fig. Structure and Hydrophobicity of E.coli SecA**


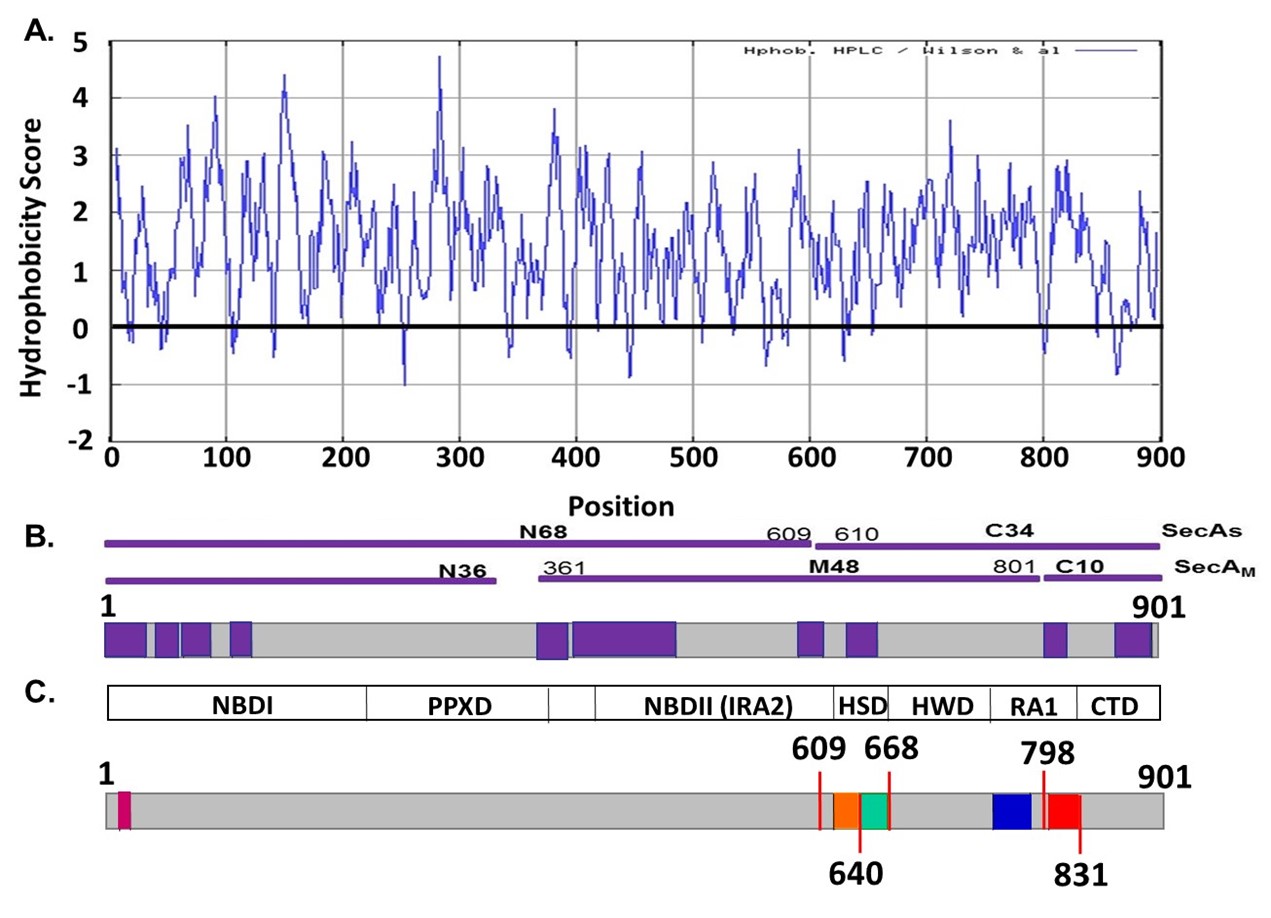


**D.**


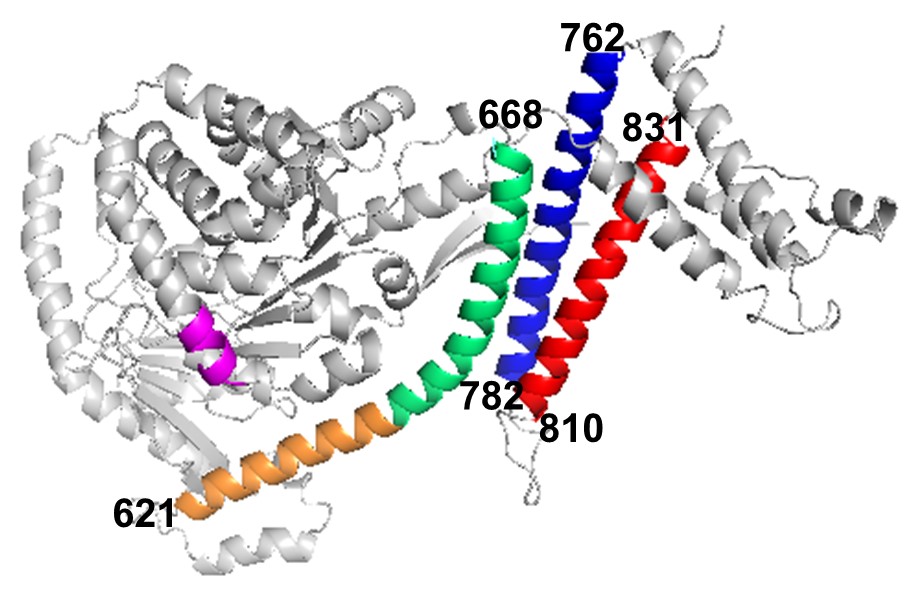

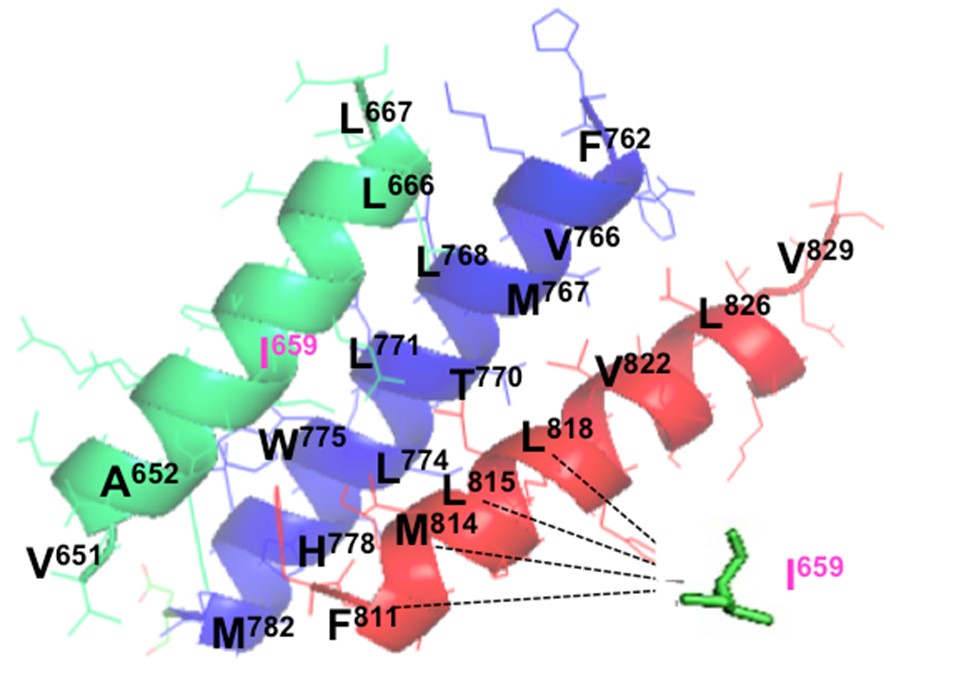


**S1 Fig. Structure and Hydrophobicity of E.coli SecA**

**A**. Hydrophilicity plot of SecA sequence [34] (from proScale, ExPASy Bioinformatics Resource Portal).

**B**. Multiple lipid-binding domains [15, 22, 25, 31, 33, 51, 52]. N68 (N609), C34 (# 610-901), N350 and M48 (SecA361-798) are trypsin fragments ; the latter 2 are lipid-specific [15, 22].The lower bars: known and predicted SecA lipid-binding domains [60]: (SecA1-21, SecA14-33, SecA43-60, SecA66-90, SecA108-125, SecA370-395, SecA400-488 SecA593-614 SecA635-660 SecA804-822 SecA865-882, SecA877-895).

**C**. Linear presentations of critical SecA domains and Subdomains: Nucleotide binding domain (NBDI and II NBDI: A1:102-109; B1:198-210; NBDII: A2:503-511B2: 631-653 [34]; Preprotein binding domain); α-helical wing domain (HWD; a long helical scaffold domain (HSD) #621-668 containing two subdomains:# 621-640 (Orange) important for forming pores and 641-668 (Green) critical for SecYEG interaction [14, 32, 64].1 deep-blue helix RA1 (# 756-788) for channel activity; 1 red-helix (#802-829) for protein translocation.

**D**. Simulated EcSecA X-ray ribbon structure [30]. (Protein Data Bank #2FSF; only #12-831 available; Pink, N-terminal helix #1-11; precursor binding domain, (PPXD) and C-terminal domains CTD are not available). E. coli SecA monomer 3D ribbon structure predicted by Pymol software (The PyMOL Molecular Graphics System, Version 1.7.4 Schrödinger, LLC.).

Left panel: SecA ribbon structure of 3 highlighted critical helix domains. The 3 helical domains #609-831 form hydrophobic interactions and are probably critical for SecA functions. Right panel: Zoom in simulated 3D structure of three critical helices with interacting hydrophobic residues. Green: 651-667aa, Blue: 762-782aa, and Red: 810-829aa. The hydrophobic amino acids are marked on three helices. The I695 is close to F811, M814, L815 and L818 after 180 degree turn on 3D structure.
